# Evaluation of swallowing function during the perioperative period using fiberoptic endoscopy in a patient with myasthenia gravis: a case report

**DOI:** 10.1186/s40981-025-00783-y

**Published:** 2025-04-02

**Authors:** Kunihiro Mitsuzawa, Takashi Ishida, Mariko Ito, Satoshi Tanaka, Mikito Kawamata

**Affiliations:** 1 Department of Anesthesiology and Resuscitology, Shinshu University School of Medicine, 3-1-1, Asahi, Matsumoto City, Nagano, 390-8621 Japan; 2 Department of Anesthesiology, Nagano Red Cross Hospital, 5-22-1, Wakasato, Nagano, 380-8582 Japan

**Keywords:** Fiberoptic endoscopic evaluation of swallowing, Swallowing function, Myasthenia gravis, General anesthesia, Endotracheal intubation

## Abstract

**Background:**

General anesthesia causes postoperative dysphagia, and myasthenia gravis also impairs swallowing function. Thus, managing general anesthesia in patients with myasthenia gravis requires special attention to swallowing function. Fiberoptic endoscopic evaluation of swallowing (FEES) has the potential to provide precise perioperative assessment and management of swallowing in these patients.

**Case presentation:**

A 35-year-old woman with myasthenia gravis was scheduled for laparoscopic ileocolic resection. FEES was performed before anesthesia, after extubation, and on postoperative day 1. General anesthesia was performed with endotracheal intubation, and extubation was performed uneventfully. Post-extubation FEES revealed salivary pooling, decreased glottal closure reflex, and redness of right arytenoid, likely caused by the endotracheal intubation and nasogastric tube. However, FEES performed on postoperative day 1 showed improvement of these findings.

**Conclusions:**

FEES effectively identified transient swallowing impairments related to intubation and confirmed the absence of dysphagia specific to myasthenia gravis, thereby contributing to safe perioperative care.

## Background

In general anesthesia, swallowing is impaired because the anesthetic itself decreases swallowing function and endotracheal intubation decreases laryngeal proprioception [1, 2]. Myasthenia gravis (MG) also causes swallowing and respiratory muscle weakness [3–5], and swallowing function impairment after general anesthesia may be more pronounced in patients with MG than in normal individuals. Therefore, patients with MG undergoing general anesthesia require careful management, particularly regarding respiratory and swallowing function. Fiberoptic endoscopic evaluation of swallowing (FEES) is a procedure that involves transnasal flexible fiberoptic endoscopy to directly visualize the pharyngeal and laryngeal structures during swallowing and FEES is considered to be a useful method for precise evaluation of swallowing function because it detects aspiration, laryngeal penetration, and pharyngeal residues with a high detection rate [6, 7]. There are few reports on the utility of FEES during the perioperative period, and changes in swallowing function in patients with MG during this period have not been clearly elucidated. We present a case report on evaluation of perioperative swallowing function using FEES in a patient with MG who had mild swallowing dysfunction. Written informed consent for publication of this case report was obtained from the patient.

## Case presentation

A 35-year-old woman (height, 167 cm; weight, 69 kg) was scheduled for laparoscopic ileocolic resection under general anesthesia due to recurrent appendicitis. She had been diagnosed with MG and had undergone thymectomy 3 years prior (Myasthenia Gravis Foundation of America clinical classification IIa). She was being treated with pyridostigmine at 270 mg/day and dexamethasone at 0.25 mg/day orally. She had ptosis and mild dysphagia, characterized by difficulty in swallowing water during fatigue, though there was no fatigue deterioration by repetitive swallowing. Results of pulmonary function tests were within normal limits. We scheduled FEES at three perioperative timings (before anesthesia, after extubation, and postoperative day (POD) 1) to guide postoperative respiratory management and to determine the optimal timing for resuming oral intake.

**Fig. 1:**
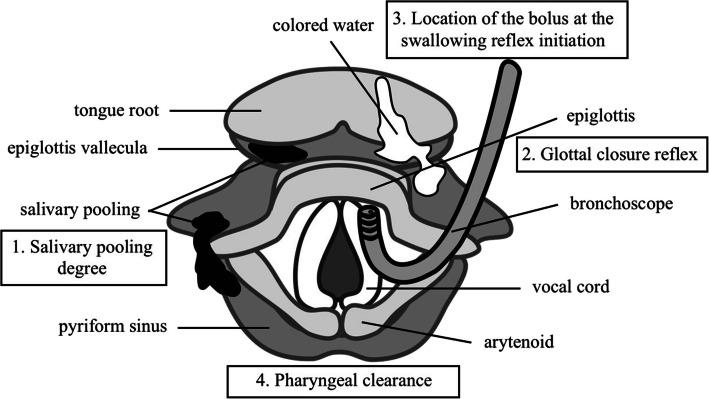
Scheme of fiberoptic endoscopic evaluation of swallowing using the Hyodo score. At first, a bronchoscope was inserted and salivary pooling (1. Salivary pooling degree) was evaluated. Then, the bronchoscope was brought into contact with the epiglottis and laryngeal sensation (2. Glottal closure reflex) was evaluated. After evaluating the sensation, 3 ml of colored water was put in her mouth and the patient was directed to hold the water in mouth to assess early inflow into the pharynx before swallowing. After the intraoral holding, the patient was directed to swallow the water and the delay of swallowing reflex initiation (3. Location of the bolus at the swallowing reflex initiation) and pharyngeal residue management (4. Pharyngeal clearance) were evaluated

**Fig. 2:**
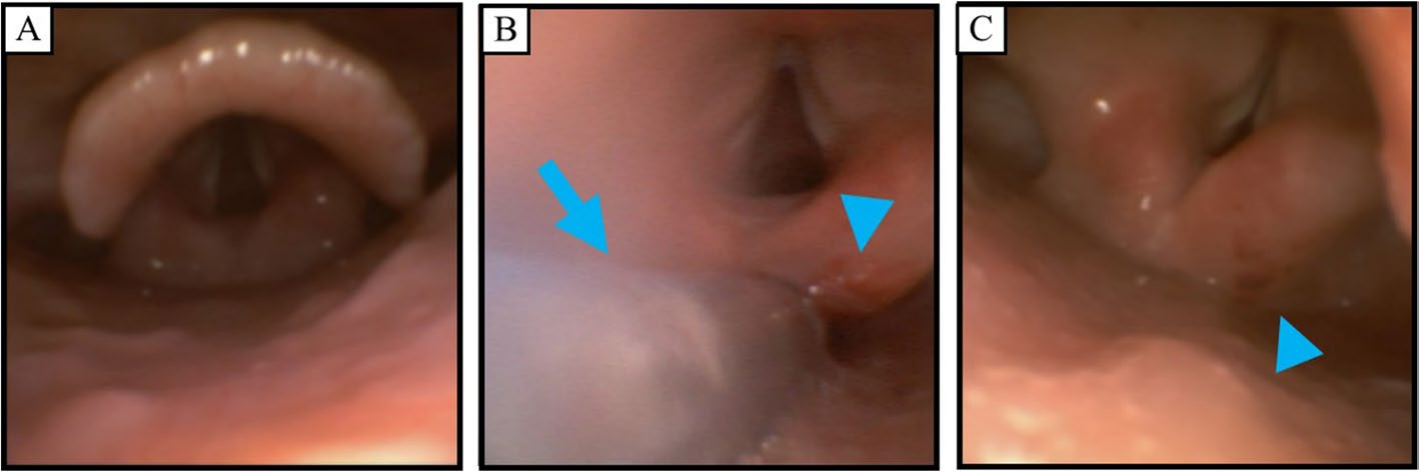
Images of fiberoptic endoscopic evaluation of swallowing (FEES). **A** FEES before anesthesia. Saliva pooling was slight and the glottis was normal. **B** Twenty minutes after extubation of the endotracheal tube (FEES after extubation). Sputum was pooling around the nasogastric tube (arrow) and the right arytenoid in contact with the nasogastric tube had redness (arrowhead). **C** Postoperative day (POD) 1 after the removal of nasogastric tube (FEES POD 1). The redness on the right arytenoid remained (arrowhead), but saliva pooling was improved to the same level as that before anesthesia

No premedication was given. Following insertion of an epidural catheter at the Th10/11 level, FEES was performed prior to general anesthesia as follows (FEES before anesthesia). The patient was positioned in a 30-degree semi-Fowler’s position with a soft pillow. A bronchoscope (Ambu® aScope™ broncho slim, Ambu, Ballerup, Denmark) was inserted nasally after nasal application of 4% lidocaine and 0.02% adrenaline. At first, salivary pooling, laryngeal penetration (food or liquid entering the larynx), aspiration, laryngeal sensation (glottal closure reflex), and other anatomical or functional disorders were evaluated. Then, 3 ml of colored water was put in the patient’s mouth and the patient was directed to hold the water in her mouth to assess early inflow into the pharynx before swallowing. After the intraoral holding, the patient was directed to swallow the water and the delay of swallowing reflex initiation and pharyngeal residue (pharyngeal clearance) were evaluated (Fig. 1). We used the Hyodo score (a 4-point scale per item, consisting of 4 evaluation items from 0 to 3, 0 meaning normal and 3 meaning worst; total score of 0–12 points) for evaluation of dysphagia [8]. The patient’s Hyodo score was 0/12 points (Table 1), indicating normal swallowing function (Fig. 2A).

**Table 1:** Scores of fiberoptic endoscopic evaluation of swallowing (Hyodo score)

	Before anesthesia	After extubation	POD 1
Salivary pooling degree	0/3	1/3	0/3
Glottal closure reflex	0/3	3/3	1/3
Location of the bolus at the swallowing reflex initiation	0/3	0/3	0/3
Pharyngeal clearance	0/3	0/3	0/3
Total score	0/12	4/12	1/12

Hyodo score is a 4-point scale consisting of 4 evaluation items from 0 to 3 (0, normal; 1, mildly impaired; 2, moderately impaired; 3, severely impaired) and a total score of 0–12 points (0 meaning normal and 12 meaning worst). All tests were performed in the 30 degrees semi-Fowler’s position with a soft pillow. The nasogastric tube was retained during the test after extubation

*POD* Postoperative day

After the FEES, general anesthesia was induced with 110 mg propofol. After confirming loss of consciousness, a sensing electrode (NM-345Y™, Nihon Kohden, Tokyo, Japan) was placed on the right adductor pollicis muscle and connected to a neuromuscular monitor (AF-201P™, Nihon Kohden, Tokyo, Japan). After calibration of the neuromuscular monitor, rocuronium was administered in five boluses of 10 mg each at 2-min intervals (total of 50 mg) until the reaction of train-of-four (TOF) stimuli diminished. The trachea was intubated with an endotracheal tube (Shiley™ TaperGuard™ endotracheal tube, Medtronic, Dublin, Ireland; 7.0 mm in inner diameter), and a 14 French sized nasogastric tube was inserted using video laryngoscopy (McGRATH™ MAC, Dublin, Ireland). General anesthesia was maintained with 5% desflurane, 0.07–0.17 μg/kg/min of remifentanil, and intermittent intravenous administration of total 300 μg of fentanyl. Rocuronium was administrated intermittently when the TOF reaction became two or more (total 60 mg). Six milliliters of 0.125% levobupivacaine was administered every 1.5 h for intraoperative epidural anesthesia, and continuous epidural anesthesia consisting of 0.125% levobupivacaine at 4 ml/h and fentanyl at 10 μg/h was started at the end of surgery.

The surgery was performed uneventfully and administration of remifentanil and desflurane was discontinued. TOF ratio was 46% at the end of surgery. After 280 mg (4 mg/kg) bolus administration of sugammadex, TOF ratio increased to over 100% (118–125%) and the trachea was extubated uneventfully with alert wakefulness and good spontaneous respiration.

Twenty minutes after extubation, she was alert and FEES was performed as previously described (FEES after extubation). Saliva and sputum accumulation around the nasogastric tube was observed, and redness was seen on the right arytenoid where the nasogastric tube was in contact (Fig. 2B). Glottal closure reflex was absent when the bronchoscope was in contact with the epiglottis. Swallowing reflex initiation and pharyngeal clearance were normal and fatigue deterioration by repetitive swallowing was not seen. The Hyodo score was 4/12 points (Table 1). Although salivary pooling and decreased glottal closure reflex were observed, evident aspiration was not seen and the protective condition from aspiration (alert wakefulness and sputum expectoration) was conserved. After FEES, she was transferred to the intensive care unit.

On POD 1, the nasogastric tube was removed and FEES was performed (FEES POD 1). Although the redness on the right arytenoid persisted, salivary pooling and decreased glottal closure reflex were improved (Fig. 2C). The Hyodo score was 1/12 points (Table 1), indicating that swallowing function had nearly returned to the preoperative baseline. After FEES, oral hydration was resumed without nausea and vomiting. She was transferred to the surgical ward on POD 1 and oral feeding was resumed on POD 3. She was discharged to home without any complications on POD 14.

## Discussion

To the best of our knowledge, this is the first report on evaluation of changes in perioperative swallowing function in a patient with MG using fiberoptic endoscopy. Dysphagia is a common postoperative complication, and it occurs in up to 43% of patients undergoing general anesthesia with endotracheal intubation [1]. Dysphagia is caused not only by mechanical injury from endotracheal tube insertion but also by residual effects of anesthetic agents and muscle relaxants [9–11]. In patients with impaired swallowing function, the effect of general anesthesia and endotracheal intubation on swallowing function would be pronounced. In such patients, dysphagia increases the risk of aspiration pneumonia and respiratory failure, potentially delaying postoperative recovery and prolonging hospital stays [2]. Therefore, to prevent these complications, we attempted to evaluate perioperative swallowing function in a patient with MG who had mild swallowing dysfunction.

FEES and videofluoroscopy are standard methods for precise evaluation of swallowing. FEES has been reported to have a higher detection rate for aspiration, laryngeal penetration, and pharyngeal residues [6, 7]. FEES also enables detection of anatomical injuries such as edema, hematoma, and vocal cord paralysis caused by endotracheal intubation, which cannot be detected by videofluoroscopy [1]. Thus, FEES is useful for precise evaluation and management of swallowing.

MG causes skeletal muscle weakness, predominantly in facial and swallowing muscles [12]. Dysphagia is an early manifestation in MG patients and eventually occurs in 40–60% of all patients [4, 5]. Even in patients without overt dysphagia, impaired swallowing function can be detected through videofluoroscopy, and silent aspiration can also be detected in MG patients with mild dysphagia [5, 13]. Aspiration is known to induce myasthenic crisis, and prevention of silent aspiration is important to avoid respiratory failure [14]. During FEES, patients with MG commonly show pharyngeal residue in the vallecular space and pyriform sinuses, which is improved significantly by administration of edrophonium [15, 16].

In this case, FEES after extubation detected the revealed function of glottal closure reflex and redness of the right arytenoid, where the nasogastric tube was in contact. Redness or edema in the larynx is commonly reported after extubation [1], and decreased glottal closure reflex is considered to be due to desensitization of the laryngopharynx by the endotracheal tube or anesthetics [2, 17]. The reason for the impaired glottal closure reflex after extubation in this case is unclear. One potential mechanism for transient impairment of glottal closure reflex is that even a small amount of residual effects of the drugs used in general anesthesia affect the swallowing function in patients with MG as mentioned above. Another possible mechanism is that the impact of endotracheal intubation or contact of the nasogastric tube around the glottis interfered with the movement of the vocal cords. It has been reported that a nasogastric tube affects swallowing function due to loss of anatomical integrity of the upper and lower esophageal sphincters, increase in frequency of transient lower esophageal sphincter relaxations, and desensitization of the pharyngoglottal adduction reflex [18]. These effects associated with a nasogastric tube increase aspiration and pharyngeal residue and prolong transit of diet and fluid through the pharynx in older healthy individuals [19]. Thus, both temporary endotracheal intubation and nasogastric tube placement may have impaired the glottal closure reflex due to laryngopharyngeal desensitization and, furthermore, the nasogastric tube may have affected swallowing function by loss of anatomical integrity of the upper and lower esophageal sphincters and increasing the pharyngeal residue. However, there has been no systematic research about perioperative FEES, and the precise time course of recovery of swallowing function after general anesthesia remains unclear. Further research on perioperative FEES is needed to establish the appropriate postoperative respiratory and swallowing management for individual patient conditions.

In this case, the pharyngeal residue during FEES after extubation was mild and improved by POD 1. Additionally, there was no fatigue deterioration by repetitive swallowing. These findings suggest that the impaired swallowing function after extubation was mainly attributable to the general anesthesia with endotracheal intubation and nasogastric tube insertion, and the impact of MG on perioperative swallowing function in this case was considered to be minimal. This assessment was instrumental in determining the appropriate timing for postoperative resumption of oral intake.

In conclusion, we reported an MG patient with mild dysphagia who underwent general anesthesia and perioperative FEES. This case highlights the potential benefits of FEES in guiding perioperative swallowing management and ensuring patient safety.

## Data Availability

Not applicable.

## Abbreviations

MG: Myasthenia gravis
FEES: Fiberoptic endoscopic evaluation of swallowing
TOF: Train of four
POD: Postoperative day
